# The sustainability of group empowerment and training for people with diabetes in South Africa

**DOI:** 10.4102/safp.v66i1.5918

**Published:** 2024-05-06

**Authors:** Robert J. Mash, Darcelle Schouw

**Affiliations:** 1 Division of Family Medicine and Primary Care, Faculty of Medicine and Health Sciences, Stellenbosch University, Cape Town, South Africa

**Keywords:** diabetes, group empowerment, group education, self-management, implementation, sustainability

## Abstract

**Background:**

Group empowerment and training (GREAT) for people with type 2 diabetes enables self-management and lifestyle modification. GREAT for diabetes was implemented in primary care facilities in five South African provinces in the beginning of 2022. The aim was to evaluate implementation and to particularly explore factors that influenced the sustainability of implementation.

**Methods:**

An exploratory, descriptive qualitative study conducted semi-structured individual interviews with 17 key stakeholders at the end of 2023. Interviews explored factors within a theory of change framework derived from an initial evaluation in 2022. Data were analysed using the framework method and ATLAS.ti.

**Results:**

Implementation and scale-up was sustained in the Western Cape. Governance and financing at a provincial and district level were key to health system structures. Space, staffing, resource materials and monitoring of implementation were key to the inputs. Facility managers, training and performance of facilitators, including the whole team, selecting patients, patient flow and appointments, stakeholder support and clinical governance were key to service delivery. Facilities that had implemented, reported reaching 300 patients per year. A range of motivational, behavioural and clinical outcomes were reported. Future implementation could include community health workers and group empowerment for insulin initiation.

**Conclusion:**

Implementation and scale-up was only sustained in one province and a range of factors related to sustained implementation were identified.

**Contribution:**

The factors identified can guide the successful implementation and scale-up of GREAT for diabetes in South Africa.

## Introduction

South Africa is experiencing an increasing problem with type 2 diabetes. Up to one in four adults over the age of 45 years has type 2 diabetes, and this increases to one in two when prediabetes is included.^1^ In South Africa, diabetes is now the second leading cause of mortality and responsible for premature deaths in many working-age adults.^2^ High rates of hospitalisation are seen for the metabolic complications as well as the many micro- and macrovascular complications.^3^ New strategic goals for noncommunicable diseases (NCDs) aim for 90% of the population to know their status, 60% of those with diabetes to receive an intervention and 50% of those to be well controlled.^4^ Current data are not able to measure the first two goals with any accuracy and suggest that in the public sector only around 25% of people with diabetes achieve glycaemic control (HbA1c < 7%).^3^

One of the key problems is a lack of patient empowerment to self-manage diabetes and make appropriate lifestyle changes. Reasons for this include a negative attitude among health workers towards the effectiveness of lifestyle counselling and a high workload with a lack of time, and brief mechanistic consultations.^5,6^ Health professionals may also have poor knowledge of lifestyle modification, language barriers and lack skills in behaviour change counselling.^5^ The health system also has far fewer resources to tackle NCDs relative to the historic priorities of human immunodeficiency virus (HIV) and tuberculosis (TB).

Group empowerment and training (GREAT) for type 2 diabetes was developed to try and overcome some of these challenges.^7,8^ Working with groups of people outside the consultation should allow a reasonable reach, as well as a comprehensive and systematic approach, while not delaying service delivery.^6^ The concept was developed in Cape Town as four sessions to address an understanding of diabetes, lifestyle modification, use of medication and avoiding complications.^9^ The content was delivered in a guiding style, derived from motivational interviewing.^10^ Training was developed and provided to healthcare workers in a 3-day workshop to facilitate the sessions. A number of studies demonstrated its feasibility and cost-effectiveness in our context.^11,12,13,14^ The next challenge was to take such an initiative to scale within public sector primary care.

**FIGURE 1:**
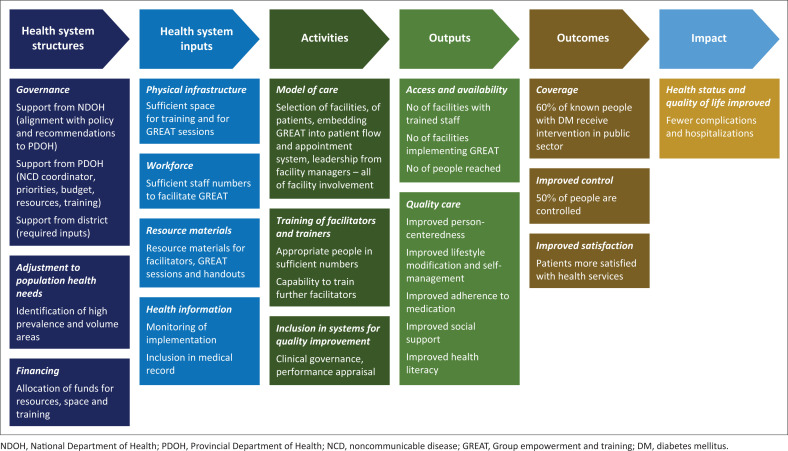
Theory of change for implementation of group empowerment and training.

The World Diabetes Foundation funded implementation of GREAT for diabetes across South Africa. The intention was to introduce GREAT for diabetes in one district in each of the nine provinces. Only five provinces agreed to collaborate, and implementation took place during 2019–2022. Implementation was severely impacted by coronavirus disease 2019 (COVID-19), as people with diabetes were at high risk and bringing groups together was impossible during the pandemic and lockdown. Despite this, implementation was restarted at the beginning of 2022 and evaluated 12 weeks later. Initial evaluation led to the creation of a theory of change (Figure 1) on the key factors that influenced successful implementation.^15^ At the end of 2023, we aimed to evaluate implementation a year later and to particularly explore factors that influenced the sustainability of implementation.

## Methods

### Study design

An exploratory, descriptive qualitative approach explored the sustainability of GREAT for diabetes.

### Setting

Group empowerment and training for diabetes was implemented in the provinces of the Northern Cape (ZF Mgcawu District), Gauteng (City of Johannesburg and Tshwane District), KwaZulu-Natal (Umgungundlowvu District) and Western Cape (Khayelitsha-Eastern Substructure [KESS]). Although GREAT was initially implemented in the province of North-West, they did not respond to requests for follow-up interviews. In all these settings, the funding supported implementation at 10 primary care facilities. During 2023, the Metro Health Services in the Western Cape also implemented in 15 primary care facilities in the Northern-Tygerberg Substructure (NTSS).

### Conceptual framework

The initial evaluation of implementation resulted in a theory of change (Figure 1) in the format of a logic model.^15^ This health systems approach categorised the key factors into health system structures and inputs that support service delivery activities and should lead to a variety of outputs, outcomes and a longer-term impact. This study revisited these issues and explored the sustainability of implementation more than a year after the initial evaluation.

### Study population, sample size and sampling

Two key informants were identified in each of the provinces where GREAT was implemented. One informant was purposefully selected from the health system structure or inputs sections of the theory of change and one from the service delivery section. The intention was to have feedback from a provincial or district level manager as well as a facility level perspective. The person responsible for NCDs at the National Department of Health was also selected. Additional informants were identified from the Metro Health Services where implementation had been most successful from the facility and substructure levels in both KESS and NTSS. A minimum of 11 interviews were planned and additional interviews continued until there was saturation of data.

### Data collection

A semi-structured interview guide was developed based on the theory of change (Figure 1). The opening question was ‘What do you think are the important factors that enabled or prevented successful implementation of GREAT for diabetes in your facility, subdistrict or province?’ Additional questions were designed to explore health system structure and inputs, facility level activities and perceptions of the effects, outputs and outcomes. Further questions were added to explore beliefs about capabilities and motivation of healthcare workers as recommended by the theoretical domains framework for implementation research.^16^ D.S. conducted and recorded interviews in English. These were conducted face-to-face in the Western Cape and via Zoom or TEAMS software in other provinces. Interviews lasted between 30 min and 60 min, during September 2023 and October 2023. Districts were also asked for any numerical data on reach.

### Data analysis

A professional transcriber created verbatim transcripts, which were checked against the recordings. Data analysis was conducted by D.S. and R.M., according to the framework method,^17^ and supported by ATLAS.ti software version 23. Analysis was somewhat deductive, in that codes arose inductively from the data, but were categorised according to the theory of change framework. The researchers were open to themes emerging that did not fit the framework. The following steps were followed:

- Familiarisation: D.S. and R.M. independently familiarised themselves with three transcripts and noted key issues that could be coded.
- Coding index: D.S. and R.M. shared the issues that they had identified and agreed on a set of defined codes that were also organised into categories.
- Coding: D.S. and R.M. independently coded half of the transcripts. Both coded transcripts from the Western Cape and two other provinces.
- Charting: The two data bundles were merged in ATLAS.ti to combine all codes and code families. Reports were created for each code family.
- Interpretation: D.S. and R.M. divided the reports between them and interpreted them for themes. The initial interpretations were then integrated by R.M. and checked by D.S.

### Ethical considerations

Ethical approval for the study was obtained from the Health Research Ethics Committee of the University of Stellenbosch (reference number: N19/09/127). Approval for the study was also obtained from the relevant provincial research committees.

## Results

Overall, 17 semi-structured interviews were conducted and included 10 stakeholders in KESS and NTSS in Western Cape, 2 stakeholders from Gauteng, Northern Cape and KwaZulu-Natal, and 1 stakeholder from the National Department of Health. The interpretation of data is presented in categories according to the theory of change (Figure 1). Table 1 presents an overview of the categories, themes and supportive quotations.

**TABLE 1:** Categories, themes and supportive quotations.

Category	Theme	Quotations
Health system structures	Governance	‘In our case, what we have done, and I think what would drive us for this year, is that we’ve actually made GREAT for diabetes part of our operational plan.’ (Manager NTSS) ‘You see, that one it will be a little bit difficult because when the program started, I was not a district manager at that time. I think I was one of the other sub-district … I also didn’t get that training. So, the then district director, I’m not sure what happened, if she understood and could support in terms of the management side.’ (NCD Manager NC) ‘I would say minimum, it is minimum support because our senior managers are the one that are actually sitting as a cluster when we agreed it is sub directorate. It was them gonna be taking the matter to a bigger cluster, but unfortunately there it is no, it has never been discussed.’ (NCD Manager KZN)
	Alignment with population needs	‘Phew! There’s more than sufficient people that are suffering from diabetes, you know, in the area. I think with our last stats we have an estimate of plus minus 3000 patients that are, you know, having diabetes.’ (Health centre KESS) ‘Yes, yes. I truly believe that there is a need and there’s … I think there’s quite a lot of patients that are diabetic, so it’s needed, it’s really a must have around here.’ (District management NC)
	Financing	‘And I think it’s a bit of a challenge. Funding would be a big challenge, because you got to procure the materials. And yes, it is … maybe not that much amount of money, but to fit it in your service priorities, it does become a bit of a challenge.’ (Manager NTSS) ‘But the last time I was speaking to them they said in the province they also don’t have money and that is why there is no district support in the province because they don’t have money.’ (Manager KZN)
Health system inputs	Physical infrastructure	‘When we started we had a problem with space, but along the way we got a community hall, but even though we had a problem with the space, we made a plan.’ (Facilitator GP) ‘I know most of our facilities, some of them they even have ablution area as a counselling … so, the space, most of them is really not sufficient.’ (NCD Manager NC) ‘We do have an issue with space but we have allocated a room now that is available on the days that we want to do groups.’ (Family physician NTSS)
	Sufficient staff to facilitate GREAT	‘Yeah, I do … I do think if maybe they delegate it to maybe your staff nurse or assistant nurse and so on, because it’s all about education. So, I think it can work that way. Maybe when they are finished doing their own observation and the professional nurse are doing the other clinical work, then they can do the GREAT for diabetic.’ (NCD manager NC) ‘We do have enough staff.’ (Facilitator GP)
	Provision of resource materials	‘The other part I think that has been very instrumental, is the fact that the people actually, from the training, they actually went away with the tools, so they are ready for implementation. We procured the material last year still, in terms of, so we were preparing actually since early 2022.’ (Deputy Director NTSS) ‘Yes, I want to believe that they still have those resources but maybe one needs to check whether are they kept in a safe space or they have developed the legs to move around.’ (NCD Director KZN)
	Monitoring of implementation	‘The implementation of GREAT and the monitoring of GREAT is not part of our regular health information system. It’s not part of regular data that we gather, so yeah. The way we have been able to track it, it’s really just by like a paper-based register showing these are the number of patients that have been attending the sessions.’ (Family physician KESS)
Service delivery	Facility management support	‘Mrs X [*substructure manager*] is a fan … sorry to mention her name, she’s a fan for GREAT, so she was really ensuring with our facility manager that we run and make sure that this is implemented and sustained in our facility.’ (Facilitator KESS) ‘So, there’s a tendency to train only the champions [*facilitators*] and then when they go back, the [*facility*] manager is not even there, she does understand or does not understand, that’s actually no good.’ (NCD coordinator KZN)
	Training of facilitators	‘We’ve got three staff members trained. It would help to have more staff members trained and the right people trained, in order for it also to be successful.’ (Family physician NTSS) ‘Yeah, and the other issue that I think was also a factor, was not everybody was trained and those who were trained, actually didn’t also share or cascaded the training that they got.’ (District manager NC)
	Whole team approach	‘Maybe it wasn’t sustained [*temporarily stopped*] because there wasn’t whole team involvement, you know? There wasn’t lots of engagement and participation with all the members.’ (Family physician KESS) ‘You know, it is a success because we are working hand in hand with our clinicians.’ (Manager GP)
	Selection of patients	‘Currently we’ve been identifying those that are struggling … [*Also*] identified those that are diagnosed. We also make sure that they are recruited to the program, so that we can give them enough education.’ (Facilitator GP) ‘Our clients are identified by the doctor … okay, number one, it’s uncontrolled diabetes and newly diagnosed clients.’ (Facilitator KESS)
	Patient flow and organisation	‘Look, patients are mainly concerned about the … how long they spend in facilities. So, the first thing I have indicated to the facilitators, implementors of GREAT is, you must work out a system where somebody is being rewarded for attending a GREAT session, so that person shouldn’t go to the back of the queue, but rather keep his place or even be promoted forward if he attends.’ (Manager NTSS)
	Facilitators	‘They’re working a bit in silo, so one team member would kind of do their own thing or plan their own thing and the other two doesn’t know what’s going on. Those are the main issues.’ (Family physician NTSS) ‘When our dietician that was trained left our facility and we didn’t have a dietician for a considerable period of time, leaving only the health promotion officer, the only one really trained. Nurses that have been trained have left, there was an officer that was trained who also left, so at one point it was just our health promotor … and you can imagine her trying alone to do this in that place where there are so many competing priorities.’ (Family physician KESS) ‘People tend to be trained, but also go back to the normal routine, go back to the [*previous*] way of doing things … which means, they will then say to you, hey, but our clinic is full, there’s a large number of clients, I do not find time to provide sessions for GREAT.’ (NCD coordinator KZN)
	Appointments and attendance	‘Because most of them [*patients*] they are working so we need to make sure that our times and our meetings are flexible that they also accommodate them.’ (Facilitator, GP) ‘It seems that in all the groups, the entire … or 80 to 90% of the group members, actually came back for their follow up sessions.’ (Manager NTSS) ‘With the weather and the safety in the areas to leave home, to come on a day that’s not your clinic day, or even to come to you clinic appointment, becomes difficult.’ (Family physician NTSS)
	Stakeholder support	‘The support you’ve been giving us [*Stellenbosch University*] is so awesome, you know. You have the regular meetings with us, checking with us, how is the program now.’ (Facilitator NTSS) ‘There were no other support.’ (Manager NC) ‘In fact for us we don’t have a lot of community group we have Diabetic South Africa which unfortunately I haven’t seen them for almost a year now.’ (Manager KZN)
	Clinical governance and quality improvement	‘So, at the initial visit, we are recording patients’ baseline results in the folder and we’re also keeping record of the patients’ baseline HbA1C and other parameters at the start … and then what we will do is once they’ve gone through the program, we will monitor them in like three or six months afterwards to see whether those things have improved and also the patients’ experience.’ (Family physician NTSS) ‘Since last year I think I included it [*in my performance agreement*], the year before I think, or last year.’ (Facilitator KESS) ‘Indirect, yes, but directly, no. Because your key performance areas maybe will say, ensure that you decrease the burden of diseases, but it doesn’t say implement GREAT.’ (Manager KZN)
Outputs, outcomes and future impact	Reach	‘So, this number, of 40 people … that we manage to reach for a period of four months, that is a drop [*in the ocean*].’ (Facilitator KESS)
	Effects on people, behaviour and clinical outcomes	‘Before the GREAT intervention, that client wouldn’t be concerned, because he will have the mindset that it’s the doctor’s problem, doctor will solve it. But now, the understanding is, I need to be worried when my sugar level is more than ten.’ (Facilitator KESS) ‘I would like to say I’m looking at Mr FE. He was … when he came here for the first time, he didn’t like his diagnosis at all. He didn’t … he was just very negative and watching him over the four sessions, was he became so excited, it’s like he embraced diabetes.’ (Facilitator NTSS) ‘The WhatsApp group [*between patients*], actually assisted me, because while I was in the taxi, I felt that I’m blacking out, then I was like thinking what’s going on. Then I reached for my phone.’ (Facilitator KESS) ‘The HbA1Cs that came down, especially the nurse, very impressed with her. She changed her whole diet, her whole lifestyle …’ (Facilitator NTSS) ‘Their HbA1cs they really dropped to their ideal that we were hoping for.’ (Facilitator KESS) ‘So, what they do, the ones that are coming, they say no, we are home over the weekend, my friend comes in [*who is working*], I will tell her, look here, or tell you, look here, I know you’re also diabetic, this is what you must do.’ (Facilitator NTSS)
	Ideas for future development	‘Yes, I think it is something that we should sustain in the future.’ (Family physician NTSS) ‘I think the best strategy potentially, would be then outside of the facility, because of all of the barriers I’ve already mentioned, but maybe going into the community.’ (Family physician KESS) ‘What you pick up with the patients that’s on insulin, they … they’ve got the fat tummy, now they inject there, the insulin was going nowhere, they don’t even know about rotating. They don’t know how to store the insulin, what do they do with their needles. If I ask them what do you do with your needles, no, I threw it in the waste.’ (Facilitator NTSS)

GREAT, group empowerment and training; NCD, noncommunicable disease.

### Health system structures

#### Governance

The National Department of Health (NDoH) supported the implementation of GREAT and saw that it was aligned with policy goals. They would like to see GREAT incorporated into the model of care for NCDs in each province, but recognised that provinces had autonomy over their own budgets and could not be forced to implement GREAT.

Provincial level managers in Northern Cape and KwaZulu-Natal espoused support for adopting GREAT, but this was not translated into sustained implementation. This may have been due to changing leadership, competing priorities and discouragement with the initial levels of implementation. In some cases, it appeared that managers had attempted to implement beyond the agreed remit. For example, in Johannesburg, we received feedback that the district had attempted to train many more facilitators without support from the GREAT team and had copied materials themselves. In Tshwane, implementation was conducted in collaboration with another university-based project that was trying to improve initiation of insulin. The GREAT team had assumed that such a collaboration would enhance implementation. In reality, the project lacked capacity to support implementation and actually hindered the facilities by retaining many of the educational resources within the university. Gauteng province indicated that they did not want to continue implementing GREAT.

In KwaZulu-Natal, we were told that managers attempted to implement in another district with the existing materials and untrained facilitators. This suggested that managers wanted to implement faster than the GREAT team was able to support, but then became discouraged when these initiatives were not sustained.

In the Metro Health Services of the Western Cape, two substructures adopted GREAT and incorporated implementation into their annual operational plans. This meant that implementation was monitored and reported on, throughout the year, and taken seriously by managers. Implementation at a single health centre in neighbouring Southern and Western Substructure (SWSS) initially struggled for support from the facility manager, as GREAT was not a priority in that substructure plan. They saw it as an individual project of the medical officer involved. High-level adoption of GREAT, formal inclusion in district goals and implementing at a feasible speed were key to successful implementation.

#### Alignment with population needs

All five provinces indicated that they had a very high prevalence of people with diabetes in their facilities and that GREAT was a solution to their problem, specifically in addressing patients with diabetes who were uncontrolled, to reduce the number of complications and premature deaths.

#### Financing

In general the availability of funds for NCDs has been less than funds for infectious diseases, such as HIV and TB. Respondents recognised that the allocation of funds for resources, space and training to implement GREAT was minimal, when compared to the cost of frequent visits, medication, and complications of diabetes. Increasing austerity measures and health budget cuts made allocation of funds to support new initiatives difficult.

Nevertheless, NTSS committed finances to print the resource materials and signed a memorandum of understanding (MOU) with the university. This was a key factor in enabling implementation. Despite commitments from other provinces to do the same, the MOU was never signed, thus preventing further training and scale-up. Reasons for these included changes in provincial leadership and complex bureaucracy.

### Health system inputs

#### Physical infrastructure

In some facilities, the issue of space was not an issue, as they had designated suitable areas for the GREAT sessions to take place. Facilities that could not accommodate the groups used public libraries or halls in close proximity to the facility. In the more rural areas, facilities had little to no space, and they made use of kitchens, parking lots, offices and even ablution facilities. In cases where space was an issue, one-on-one sessions were held, until they found a solution. Different services might compete for space and priority. For example, in one facility, GREAT was stopped because the SASSA (South African Social Security Agency) needed to deal with a backlog of applications and took over the space.

#### Sufficient staff to facilitate group empowerment and training

Staff in the facilities were overstretched to take care of the needs of the patients. Most facilities sent 2–3 people to train as facilitators, although not always appropriate people (see subsection on facilitators). The impression was that small rural facilities might struggle to accommodate GREAT when they had a very small workforce.

#### Provision of resource materials

The facilities in all the provinces had enough resource materials to conduct the GREAT sessions and were therefore equipped to implement. Resource materials were provided to all the staff who attended the GREAT training (10 facilities per province). Further scale-up, however, depended on the Department of Health signing an MOU to print additional resources. Only the NTSS in the Western Cape was able to do this.

#### Monitoring of implementation

According to the NDoH, the health information system for NCDs is not functioning well. It does not give the department the basic information they need to monitor strategic goals, and therefore to include GREAT was not seen as feasible at present. Districts were asked to capture data on number of groups and patients reached. However, only KESS and NTSS appeared to have collected such data. In NTSS, an indicator was officially added to the substructure dashboard. In some facilities, the clinicians informally monitored and reported on changes in HbA1c (glycosylated haemoglobin) and blood pressure. Negotiating for inclusion of a formal indicator at district level, which would be monitored by the Director, should be included in the preparation of implementation.

### Service delivery

#### Facility management support

Prior to the implementation of GREAT for diabetes, many managers and staff were sceptical on how this would impact their already busy workloads and whether the staff would have capacity to implement the programme. In NTSS, the management was motivated by their neighbouring substructure, who had already implemented and gave feedback on the positive benefits.

In some facilities and provinces, the management and staff supported the trained facilitators in their implementation, and staff had a renewed sense of energy, motivation and excitement. They saw GREAT as an opportunity to reduce their workload in the long term, as patients with improved diabetic control did not need to attend so often. In other provinces, where there was less management support and buy-in, facilitators were not motivated enough to implement GREAT and felt like it was adding to their workload.

Clear adoption by district or substructure management was important as well as practical support from the facility manager. Facilities varied in the perceived levels of support from district managers. In one province, although district level managers agreed to implement GREAT, this commitment did not percolate down to the facilities. Facility managers needed to understand the GREAT programme and be orientated towards it in order to offer support. They should have their own workshop or orientation session and attending some of the training could also help them to understand the programme.

Managers needed to monitor implementation on a regular basis and to identify gaps or challenges to implementation with a problem-solving approach. Several facilities temporarily stopped implementation over challenges, such as the space being used by another organisation or loss of trained facilitators. These roadblocks required a proactive problem-solving approach in the context of seeing GREAT as a priority. Otherwise, significant, but surmountable, roadblocks could quickly lead to abandonment of the initiative.

Implementation needed to be owned by the whole clinical team and not regarded as one person’s initiative or project. The initiative needed to become institutionalised and not regarded as just another pilot project. Managers needed to keep GREAT visible by discussing implementation regularly. Giving feedback on the benefits was also motivational, particularly when substantial improvements in HbA1c were documented. A combination of mechanisms, on the one hand, to hold people accountable for implementing plans while, on the other hand, giving feedback on the benefits worked well. Accountability was enhanced by including one’s role in implementing GREAT in individual performance agreements. Many respondents had already had this done. Sometimes performance was only indirectly appraised, for example, when the focus was on outcomes (e.g. improving glycaemic control or adherence) rather than actual activities (e.g. number of sessions facilitated).

#### Training of facilitators

In NTSS, they covered all 15 PHC facilities by running three training workshops with three people from each of the facilities. Training was provided in house by the Department of Health – three trainers were dieticians, and one was a diabetes educator from the tertiary hospital. All were released from their usual duties to assist with training. Substructures were reluctant to release staff to assist other substructures. There was a clear need to develop training capacity for the whole district through formal structures such as the People Development Centre (PDC). It was also clear that training of facilitators would be an ongoing need to sustain the initiative. The PDC, however, had their own priorities and preferred virtual online training courses. In some provinces, further training required commitment to print the resource materials and sign an MOU to enable this. This became a stumbling block to further training in these provinces as the university had no further funds to provide additional resource materials.

The style of the training programme alongside support from staff at the facility made the facilitators feel empowered, motivated and more knowledgeable to implement GREAT in their facilities. Nearly all the management, staff and facilitators across the facilities felt that they had good knowledge of diabetes, and the GREAT training had equipped staff with the necessary information. Where they felt they needed more expertise, they would learn from each other, or the clinicians would support them.

#### Whole team approach

Implementation required different roles and contributions, from receptionists to draw the folders, facilitators (who could be health promotion officers, nurses, dieticians, etc.), enrolled nurses in the preparation areas, clinicians (nurse practitioners or medical officers) to identify patients and consult patients, and pharmacy staff (to fast-track medication). The team was empowered by the family physician and facility management. Sometimes it was useful to identify a clear champion. Ownership by the whole team was therefore vital to success. Most of the facilities felt that the staff had a positive attitude and belief towards patient education and counselling in general.

#### Selection of patients

Respondents agreed that the programme should focus on people with diabetes who were uncontrolled or newly diagnosed. In some cases, patients with poor diabetic control were defined quite loosely as those ‘struggling’ or ‘not doing well’, while in other places they were defined by their HbA1c. In these instances, it was the very uncontrolled who were recruited, with HbA1c > 10% or even > 12%. Patients were selected by the clinicians and referred to the facilitator for a date to attend the first session. In one facility, the facilitator then had a pre-GREAT discussion with the patient. In one setting, they also included those with pre-diabetes. In one or two facilities, they also intended to allow staff to attend sessions. Not only could they personally benefit, but also they could share their new knowledge with patients.

#### Patient flow and organisation

**FIGURE 2:**
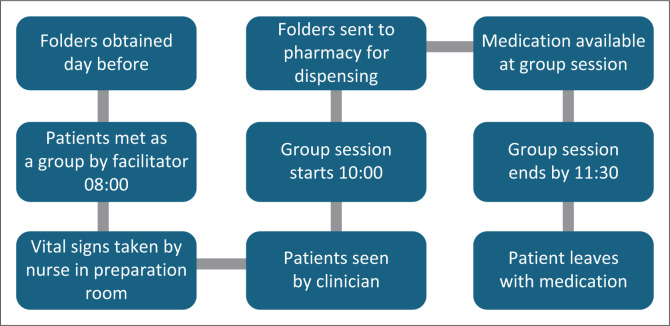
Example of patient flow through the facility.

The key principle was not to penalise people for attending GREAT by making their visit longer than others or longer than the ideal clinic norms and standards. Ideally, they should be rewarded or prioritised in some way. At the same time, the process should not adversely impact on other patients. One facility that ran sessions on the same day as usual attendance described their approach in detail in Figure 2. If this was an additional visit, then the consultation with the clinician would be omitted. One facility combined the visit to the clinician with the actual session itself.

#### Facilitators

Training of facilitators in NTSS appeared to go well with 2–3 people appropriately selected and trained from each facility. In other locations, although multiple people were trained from each facility, the number actually available to implement often dwindled quickly. For example, trained facilitators might leave the facility, be rotated to other duties (e.g. nurse practitioners), go on leave, not really be available due to their role (e.g. medical officers who must consult patients or dieticians who have several facilities to visit), have a limited focus of expertise (e.g. pharmacy assistant) or not be capable of facilitating well (e.g. retains very directive communication style). Some facilitators might also lack motivation or agency to implement.

This highlighted a number of lessons. Firstly, it was important to send the right people to the training course and, secondly, ongoing training of additional staff members will always be needed. Health promotion officers and nurse practitioners appeared to be well suited to facilitate the sessions. In one facility, the facilitator attempted to informally equip others to assist with facilitation. Patients appreciated continuity with the same facilitators when attending sessions. Where there were multiple facilitators and other staff members involved in implementation, it was important to meet regularly and plan when, where and who would be involved and to foster open communication.

#### Appointments and attendance

Although the training recommended holding the sessions on the day of the usual appointment, many facilities required patients to make additional visits for the sessions. Extra visits placed an increased burden on patients in terms of travel costs and this could impact on attendance. Inclement weather and concerns about safety could also impact on attendance on any given day. Despite this, NTSS reported 80% – 90% attendance rates.

Group empowerment and training sessions were held during office hours and were not convenient for people working. Many of the employed patients were on a ‘no work no pay’ basis and without the benefit of sick leave. Nevertheless, facilities could issue employed people with a sick certificate for the day of attendance, which might help. Greater flexibility and innovation in terms of access for employed patients was needed.

One facility reported that they had to motivate patients to attend the first session, but after that they were self-motivated to attend. Some facilities tried to combine sessions into two visits to improve attendance, but others felt that this placed too much of a burden on patients to cope with the increased content. Numbers attending the groups could vary from 5 up to 25 people, but initial group size was typically around 15 people.

#### Stakeholder support

A number of stakeholders were important to the sustainability of implementation. Many facilities recognised the value of support from family medicine at Stellenbosch University and the people who trained the facilitators. Support included checking up on progress and helping to troubleshoot around any roadblocks. One facility saw that medical students could also assist with implementation during their rotations, for example, following up with patients. In KESS, they were successful at obtaining a grant from the Discovery Foundation to support the provision of resources, training and implementation. Some facilities saw the importance of informing the clinic health committees about the initiative as they could be supportive and might receive feedback from patients.

Some respondents saw the potential of engaging with other stakeholders. For example, community health workers could benefit from attending GREAT. This might benefit them personally if they had diabetes or risk factors, but could also empower them to reinforce GREAT in the community or share the key messages with those at risk of diabetes. This informal dissemination and reinforcement could occur in households, support groups or even wellness centres. One facility reflected on whether traditional healers might benefit from attending GREAT. In KESS, there was also collaboration with a project that was providing retinal screening.

#### Clinical governance and quality improvement processes

Some facilities were already performing regular audit and feedback of the quality of care for diabetes. The effect of GREAT on quality of care outcomes (i.e. glycaemic and blood pressure control, body mass index) could easily be included in such audits. In other facilities, the clinicians were monitoring glycaemic and blood pressure control before and several months after attending GREAT and giving feedback to the team.

### Outputs, outcomes and future impact

#### Reach

Only NTSS had managed to share clear data on reach for all facilities. This may be because more effort was put into selecting and monitoring an indicator with the substructure management team. In NTSS, 11 out of 15 facilities had implemented by the end of 2023 and had reached 41 groups with 252 patients. In KESS, which had implemented the previous year, feedback from three health centres suggested that they were running two groups per month with approximately 15 patients per group. They should therefore reach 300 patients per year per facility. Facilities initially implemented slowly to ensure the first groups worked, with the intention of increasing reach later. It appeared that in the Northern Cape, implementation was not successful. In Gauteng and KwaZulu-Natal, only a handful of facilities had managed to sustain implementation. No data on reach was available.

#### Effects on people, behaviour and clinical outcomes

All respondents reported positive benefits to their patients. Group empowerment and training increased people’s knowledge and understanding of their condition and gave them insight into why their glucose went up or down. For many, this was new information despite having diabetes for more than a decade. Beyond knowledge, however, GREAT also had motivational and psychological benefits. Some patients were able to accept their diagnosis and shift their locus of control from a sense of fatalism or externalising responsibility to healthcare workers, to a sense of self-control and agency. Having greater health literacy with regard to their diabetes also enhanced the quality of interactions with clinicians.

Being part of a group was important for many. Group members were able to share relevant and context-appropriate solutions and lifestyle changes. Support continued outside the sessions, with interactions in the community or via WhatsApp groups. One patient recounted how her group helped her to cope via WhatsApp when she had hypoglycaemia in a taxi to the Eastern Cape.

Patients felt more satisfied with healthcare and more positive about their relationships with their healthcare workers. Healthcare workers felt more approachable and likely to be helpful. Clients were more motivated to utilise all the services available.

Patients also disseminated their knowledge and behaviour changes to family members and friends, and some made a special effort with those who were working and could not attend sessions. Some also spread the word that people with diabetes should attend the GREAT sessions.

Many respondents reported significant improvements in HbA1c, blood pressure and loss of weight. One facility documented substantial improvements in HbA1c among group members.

#### Ideas for future development

Respondents agreed that GREAT should be sustained as it was working for patients. A number of suggestions were made for future development:

- Provide additional health promotion materials to reinforce key messages for patients.
- Give attention to the difficulties with starting and using insulin correctly.
- Expand the implementation of GREAT into community-based services.

## Discussion

The key findings reinforce the importance of the factors identified in the initial study and the validity of the theory of change as a guide to successful implementation.^15^ Key factors were related to health system structures (provincial and district level governance, population health needs and financing), health system inputs (physical infrastructure, sufficient staff, provision of resource materials, monitoring of implementation) and service delivery activities (support of facility manager, training of appropriate facilitators, involvement of whole clinical team, selection of patients, patient flow and appointments, facilitator performance, stakeholder support and inclusion in clinical governance). It appeared that successful facilities were reaching approximately 300 patients per year. Respondents reported on a range of positive motivational, behavioural and clinical outcomes. Respondents thought that GREAT could be enhanced by having supportive patient educational materials, extending into the community and via community health workers, and also targeting people who need to start insulin. The key findings are discussed in relationship to the health system issues and the service delivery issues.

Although the national department sets policy, prioritisation within the budget, goal setting and support of innovations is decided on at a provincial level in South Africa.^18^ Alignment with national policy is important, but actual implementation requires engagement and commitment from nine different provinces. The increasing traction and success in the Western Cape was in contrast to the challenges of sustaining implementation elsewhere. The value of local relationships with district management and prior exposure to the concept of group empowerment in the Metro Health Services^8,14^ may have been critical. Relationships had developed over years, with a degree of trust and ability to engage with managers in a variety of contexts. Managers were also willing to include GREAT in district level planning and priorities and to sign an agreement with the university to print the resource materials. Implementers from the university were invited to participate in such planning meetings and to share evidence on GREAT at research days. In the NTSS, managers were willing to agree on a local indicator to monitor implementation and to integrate activities into individual performance management. This signalled to facilities that implementation was expected and would be monitored by district level managers.

Although provinces were under financial constraints during this period,^19^ the printing of resource materials was more limited by bureaucratic processes than availability of funds. Provinces seemed unable or unwilling to sign an MOU with the university to enable them to print the resources for themselves. There was an impression that the MOU became lost in the bureaucracy, was not prioritised or was seen as losing control over resources – the agreement was intended to protect the intellectual property and integrity of the materials.

At the facility level, the importance of active engagement with facility level managers both before and during implementation was exemplified by scale-up in NTSS. Appreciating and prioritising support of self-management may help to overcome a sense of futility among healthcare workers.^5^ Ten key questions were identified for facility managers to consider in planning (Table 2). Anticipating and planning for these issues in advance may be key to successful implementation; for example, ensuring that suitable space was available, that people trained as facilitators would actually be able to incorporate this into their work, that patients were not disadvantaged and that the whole team was involved. Competency in experimentation, innovation, planning and troubleshooting has been identified as a weak part of the organisational culture.^20^ The need to shift from a command and control to more collaborative styles of leadership has also been noted.^21^

**TABLE 2:** Key questions for facility managers in implementing group empowerment and training.

Number	Question
1	Which patients will you target?
2	Where will you have enough space for the groups to meet?
3	How will you embed GREAT into the patient flow – and not disincentivise patients?
4	How will you embed GREAT into the patient’s follow-up appointments to ensure they can attend all the sessions?
5	Who will lead or champion the initiative in each facility?
6	Who will be trained to facilitate the sessions?
7	Who else will be involved in implementation – clinicians, receptionists, pharmacy, etc?
8	What data will be collected to monitor implementation?
9	How will referral/attendance be noted in the medical record?
10	How will this be embedded in individual performance management?

GREAT, group empowerment and training.

Support in planning, clinical governance and troubleshooting from the family physicians was also important. Family physicians understood the need for more effective management of people with diabetes and the importance of patient empowerment. In addition, their training in leadership and clinical governance could help with successful planning, implementation and collection of local evidence of effectiveness that reinforces motivation.^22^ Going forward, it may help other provinces to actively involve family physicians and local departments of family medicine.

The study had a number of limitations. Some key informants were reluctant to be interviewed again, for example, in North-West province. We tried to balance key informants from the health system and from service delivery in order to get a balanced view of the challenges and successes. At the end of the 17 interviews, there were no new issues emerging and we believe that data saturation was achieved. It is possible that taking our more deductive approach could have overlooked issues that did not fit into the framework, although we were open to new issues emerging. Most districts had very little data on the number of patients reached or groups.

A number of recommendations can be made from this study for future implementation. Primarily, it is clear that further scale-up in the Western Cape will be easier than in other provinces. We hope to implement throughout the entire metropole and extend into the rural health services. We will continue to work with other provinces that show a desire to implement, although this may require further funding.

There is potential in extending GREAT to community-based services. Including community health workers in the GREAT programme may assist them as individuals with diabetes or at risk of diabetes, but should also empower them to share key messages with community members. This may also start to address those at risk of diabetes and contribute to disease prevention. They may reinforce self-management and lifestyle change for people with diabetes during household visits. Community health workers are recognised as a key role player in providing services for people with stable chronic conditions in the community.^23^ Community-based services also run adherence and support groups for people with diabetes that are better controlled. Sharing the sessions in this context may increase the reach of GREAT as all people with diabetes could benefit from the programme.

Access to healthcare for working people, particularly those without benefits, is a general issue.^24^ Thought needs to be given to how GREAT can be made accessible to this group of people outside of normal office hours. Community-based initiatives may assist here in addition to ensuring that certificates are offered to those with benefit of sick leave.

Finally, control of diabetes also requires initiation of insulin for many patients. This is another roadblock on the journey to better control and a recent study highlighted many of the factors involved.^25^ This study also recommended that a similar group process be developed and evaluated for people that need to start insulin. This would follow similar principles to GREAT for diabetes and run over three sessions. The materials for this have been developed and should be piloted and evaluated further.

## Conclusion

Group empowerment and training for diabetes was sustainable and had the ability to scale-up in the Western Cape, while its sustainability in other provinces was poor. The proximity of the university-based implementers and their stronger relationships with district level managers in this setting may have enabled this, along with clear prioritisation of patient empowerment for diabetes by the health services. The key factors that influence implementation in the theory of change were further validated as important to sustainability, particularly the importance of engaging and motivating facility managers and anticipating the challenges to planning and implementation. Family medicine and family physicians can be key supporters of implementation. Looking forward, there may be opportunities to extend GREAT for diabetes into the community to support patients further, include those better controlled and contribute to disease prevention. There may also be an opportunity for a similar programme to support initiation of insulin.
